# Drug Nanocarriers for Pharmaceutical Applications

**DOI:** 10.3390/pharmaceutics17080960

**Published:** 2025-07-25

**Authors:** Elia M. Grueso, Rosa M. Giráldez-Pérez, Rafael Prado-Gotor

**Affiliations:** 1 Department of Physical Chemistry, University of Seville, 41012 Seville, Spain; pradogotor@us.es; 2 Department of Cellular Biology, Physiology and Immunology, University of Córdoba, 14014 Córdoba, Spain; rgiraldez@uco.es

## 1. Introduction

Nanotransporters occupy a prominent place in medicine within the field of drug delivery and pharmaceutical applications [1]. In addition, in terms of their physicochemical properties, they have decreased toxicity compared to free drugs and facilitate specific site delivery and controlled release, effectively decreasing side effects in patients. For instance, the physicochemical properties of lipid nanoparticles (LNPs) influence the bio-nano interactions and biological effects induced. Among these properties, size, type, and value of the surface charge, as well as surface hydrophobicity and lipid composition, are particularly important and must therefore be studied in depth in each nanosystem [2]. For metallic nanoparticles (MNPs), it is vitally important to determine and control the physicochemical characteristics of their surfaces and cores to determine possible harmful consequences of their use as medicine [3]. Living organisms, including humans, are continuously exposed to inorganic nanoparticles that enter the body through various routes, such as ingestion, inhalation, penetration through the skin or blood circulation, and translocation to organs and tissues, depending on their physicochemical properties [4].

Colloidal drug transport systems can improve relevant aspects of pharmaceutical formulations such as their pharmacokinetics, biodistribution, biocompatibility, solubility, and stability [5]. In general, the solubility of a free drug is limited by its inherent chemical properties, as well as the characteristics of the surrounding environment. A fundamental problem for many drugs is their low solubility in aqueous media, as well as in the bloodstream. All of the above leads to low bioavailability [6]. In fact, more than 70,000 new molecular entities synthesized annually by pharmaceutical companies have poor solubility in aqueous media [7]. In this regard, nanotechnology stands out as a strategy for improving the solubility of these new drugs due to its ability to manipulate and modify materials at the molecular and atomic levels [8]. Furthermore, improving the solubility of drugs through the use of nanocarriers enhances their absorption, allowing more predictable pharmacokinetic profiles and a greater therapeutic effect to be achieved [9].

Another important factor to control is the effectiveness of the release of different drugs in nanomedicine, both in vitro and in vivo, since the efficacy, safety, and dosage of drugs incorporated into nanoparticles depend on this evaluation. In fact, drug release mechanisms directly influence their efficacy [10]. In this sense, nanoparticles represent a major advance in drug delivery systems (DDSs) by addressing fundamental challenges related to the ability of drugs to cross biological barriers, their biocompatibility and permeability, their degradation, and their toxicity as free drugs [11].

Over the past two years, numerous articles on various topics related to using drug nanocarriers for drug delivery and pharmaceutical applications have been published, some of which are described below. Sheng et al. designed and developed new ionic co-aggregates (ICAs) based on supra-amphiphilic compounds composed of fatty acids and choline to transport paclitaxel (PTX), a poorly soluble drug that is administered intravenously, which increased its solubility 10-fold [12]. However, a better understanding of the mechanisms responsible for the self-assembly processes involved in the formation of ICAs in aqueous solutions is needed to optimize the efficacy of the nanocomplex. Trivedi et al. examined the strategy of using amphiphilic niosomes to encapsulate both hydrophilic and lipophilic drugs [13]. These nanocarriers, about 200 nm of size, were demonstrated to be useful for tumor delivery of different anti-cancer drugs like methotrexate and doxorubicin, improving both the drug delivery and tumoricidal efficacy. In another recent study, liposome-based nanosystems were developed to transport the drug cisplatin, exploring how to optimize the loading based on varying the composition of IS and the liposomes. In this study, the DSPE-PEG/DSPG/HSPC formulation at a ratio of 10/35/55 provided the most promising results [14]. Equally, Raeispour et al. developed nanocarriers based on carbon quantum dots (CQDs) through hydrothermal synthesis to improve the therapeutic efficacy of mitoxantrone (MTX), a drug used in standard cancer therapy [15]. A drug loading efficiency of 97% was obtained, and maximal inhibition of the growth of MCF-7 cancer cells occurred after 5 h of treatment, demonstrating its high therapeutic potential. In another recent study conducted by Szczepanowicz and colleagues, new polymeric theranostic nanocarriers were synthesised using the solvent evaporation self-emulsification (SESE) method, combined with a layer-by-layer approach. The designed nanoparticles, which were a 100–120 nm diameter in size, showed calcineurin, ciclosporin A, and tacrolimus inhibition and were proven to be effective as neuroprotective agents [16].

## 2. An Overview of Published Articles

This Special Issue, entitled “Drug Nanocarriers for Pharmaceutical Applications”, aims to discuss all of the key factors to consider when preparing suitable nanocarriers for drug delivery and their use to benefit human health. Specifically, six original research articles, two of which were reviews, were published by authors from different countries on these current research issues.

Meinhard et al. (contribution 1) designed and prepared T14diLys/DOPE lipoplexes with characteristic laminar structures observed through TEM. These structures were obtained by combining the ionizable lipid 2-tetradecylhexadecanoic acid-(2-bis{[2-(2,6-diamino-1 -oxohexyl)amino]ethyl}aminoethyl)-amide (T14diLys) and the cationic gemini surfactant 1,2-dioleoyl-sn-glycero-3-phosphoethanolamine (DOPE) at different molar ratios to obtain the optimal nanocarriers for RNAi delivery. Their results showed that the complex synthesized using a nitrogen/phosphate (N/P) ratio of 5 was the best nanoformulation for transporting the biopolymer based on its optimal physicochemical and cytotoxicity properties and its encapsulation efficiency. In particular, this specific formulation resulted in small positive particles with a diameter of 162.3 nm, a narrow size distribution, and a high zeta potential value of around +40 mV, making them an optimal lipoplex for siRNA–polymer interactions. Furthermore, lipid mixture 5 (N/P) showed the highest inhibition and encapsulation efficiency of 98.8%, along with a minimum viability of 66.7% against 3T3 cells, confirming its potential for use as an optimal nanosystem for local siRNA delivery.

Meanwhile, Lee, S. et al. (contribution 2) designed and synthesized a new nanocomplex based on the combination of two species, polyethyleneimine 2k (HECP2k polymer) modified with hydroxyethylcellulose (HEC) and a nuclear factor k-B receptor activator (RANK), at an appropriate mixing ratio (1:10 *v*/*v*). This nanocomplex overcame limitations in the siRNA biopolymer and zoledronate (Zol) related to their low transfection and bioavailability. The resulting HEP2k/(RANK siRNA + Zol) nanocomplex had the optimal cell absorption properties, as verified through cytometry experiments, due to its excellent physicochemical characteristics: a small size between 140 and 180 nm, a spherical morphology, and a positive charge ranging from 17 to 24 mV. In addition, the authors studied the cell viability of the nanocomplex in both leukemic mouse monocyte–macrophage cell lines and human cervical adenocarcinoma cell lines, finding viabilities above 90%. These results, together with the high transfection efficiency ascertained in the luciferase transgene assay, confirm its low cytotoxicity and optimal transfection efficiency. It is important to note that this stable nanoformulation is intended for pharmaceutical applications to the treatment of osteoporosis, which is carried out through osteoclastic inhibition.

Velho et al. (contribution 3) present two strategies for loading ivermectin (IVM), a drug widely used to treat parasitic infections. One is loading IVM into mesoporous silica nanomaterials, and another involves IVM-loaded poly(ε-caprolactone) nanocapsules, which show a uniform nanometer size distribution and a high encapsulation efficiency. Both nanoencapsulated forms improve the intrinsic aqueous solubility of IVM compared to that of crystalline IVM: after 72 h, IVM-MCM and IVM-NC achieve 72% and 78% release through a dialysis bag, whereas the crystalline IVM dispersion achieves only 40% drug diffusion. Both nanocarriers present promising advantages in improving IVM’s dissolution rate, while this study reports the use of silica nanomaterials as carriers for IVM delivery for the first time, with potential pharmacological applications.

Francis et al. (contribution 4) formulated and evaluated a red clay (RC) nanodrug delivery system using sucrose stearate (SS) (an F1 nanocomplex) for immediate drug release in the treatment of melanoma, loading the system with acyclovir (ACV), the preferred antiviral drug for the treatment of herpes virus infections. Thus, using the immediate drug release system developed for the treatment of melanoma, cytotoxicity experiments performed on SK-MEL-3 melanoma cell lines showed that the ACV release from the F1 nanocomplex formulation inhibited melanoma cell growth with an IC50 of 25 ± 0.09 µg/mL and caused significant cytotoxicity at approximately 20 µg/mL, indicating its potential reuse for the treatment of skin cancer. These clay-based nanodrug delivery systems would also be effective for this type of cancer treatment, with minimal side effects compared to those caused by conventional chemotherapeutic agents.

Syahputra et al. (contribution 5) reviewed the current challenges and advances in specialized drug delivery approaches designed to bolster the pharmacological performance of PROTACs. PROTACs (proteolysis-targeted chimeras) are molecular tools that facilitate targeted, controlled protein degradation by recognizing and binding specific proteins through the cell’s protein degradation mechanism. This strategy offers great potential for treating common diseases such as cancer, as it reduces the accumulation of harmful proteins. These authors analyze formulations including polymeric micelles, emulsions, amorphous solid dispersions, lipid-based nanoparticles, liposomes, and exosomes and provide insights into emerging trends and the future trajectory of PROTAC-based therapies. However, they also consider different obstacles to the optimal implementation of PROTACs, such as low aqueous solubility, poor cell permeability, off-target toxicity, and hook effects, and responsive strategies.

Taheri et al. (contribution 6), in reviewing the field of stimuli-responsive nanomaterials, focus on stimuli-responsive nanocarriers functionalized using arginine–glycine–aspartic acid (Arg-Gly-Asp) sequences, as well as mimetic sequences capable of recognizing integrin receptors. The authors describe integrins as transmembrane receptors and then consider both endogenous and exogenous stimuli. In this sense, they conduct a detailed review of different influencing factors such as pH, enzyme, ROS concentration, glutathione (GSH) concentration, temperature, UV and NIR light, and magnetism. The use of integrin-targeted stimuli-responsive nanomaterials in the field of theranostics is then described. Finally, taking into account future perspectives and challenges, they consider both the factors associated with the integrin ligand and the density and degree of conjugation, which have a considerable impact not only on the physicochemical properties of theranostic agents but also their pharmacokinetic characteristics.

## 3. Conclusions and Future Perspectives

Numerous studies have demonstrated multiple organic and inorganic nanosystems that are highly effective in transporting drugs for different medical applications, improving their efficacy, biodistribution, and biocompatibility. Those included in this Special Issue demonstrate the importance of exploring the molar relationships between the components of a nanosystem, as well as the degree of conjugation and interactions among them, in order to optimize its physicochemical properties and thus its pharmacokinetic characteristics. In addition, a greater encapsulation efficiency is increasingly being achieved for different drugs, exceeding 90% in most of the cases analyzed. All of this is accompanied by high release rates of around 80% and high viability in different cell lines, generally exceeding 80%, collectively reducing the side effects of different therapies. However, several research gaps were detected in this set of contributions, especially related to effective control over global charge and size and how these physicochemical characteristics affect nanoformulations’ stability over time. Furthermore, transporting multiple doses of a drug in the same carrier is worth exploring further in order to achieve even greater therapeutic effectiveness. This could be coupled with the approach from previous studies to optimizing the molar ratio of the drug to the nanocarrier.

We would like to thank all of the authors and reviewers of this Special Issue, and naturally, we also wish to acknowledge the Assistant Editor, Ryan Pei, for his constant support and help in moving this issue forward.
